# The Relations among Fuzzy *t*-Filters on Residuated Lattices

**DOI:** 10.1155/2014/894346

**Published:** 2014-10-20

**Authors:** Huarong Zhang, Qingguo Li

**Affiliations:** ^1^College of Mathematics and Econometrics, Hunan University, Changsha, Hunan 410082, China; ^2^Department of Mathematics, China Jiliang University, Hangzhou, Zhejiang 310018, China

## Abstract

We give the simple general principle of studying the relations among fuzzy *t*-filters on residuated lattices. Using the general principle, we can easily determine the relations among fuzzy *t*-filters on different logical algebras.

## 1. Introduction

Residuated lattices, invented by Ward and Dilworth [1], constitute the semantics of Höhle's Monoidal Logic [2]. Residuated lattices are very useful and are basic algebraic structures. Many logical algebras, such as Boolean algebras, MV-algebras, BL-algebras, MTL-algebras, Gödel algebras, NM algebras, and R0-algebras, are particular residuated lattices. Besides their logical interest, residuated lattices have lots of interesting properties. In [3], Idziak proved that the varieties of residuated lattices are equational.

Filters play a vital role in investigating logical algebras and the completeness of the corresponding nonclassical logics. From logical points of view, filters correspond to sets of provable formulae. At present, the filter theory on different logical algebras has been widely studied. Only on residuated lattices, such literatures are as follows: [4–11]. In [8, 9], Ma et al. found the common features of filters on residuated lattices. They, respectively, proposed the notion of *τ*-filters and *t*-filters on residuated lattices. In [9], Víta studied some basic properties of *t*-filters and gave the simple general framework of special types of filters.

After Zadeh [12] proposed the theory of fuzzy sets, it has been applied to many branches in mathematics. The fuzzification of the filters was originated in 1995 [13]. Subsequently, a large amount of papers about special types of fuzzy filters was published in many journals on different logical algebras [10, 11, 14–24]. In [23], Víta found the common features of fuzzy filters on residuated lattices. He proposed the notion of fuzzy *t*-filters and proved its basic properties. However, the relations among fuzzy *t*-filters were not discussed. Usually, when studying the relations among special types of fuzzy filters, the equivalent characterizations of special types of fuzzy filters were firstly discussed. Then, resorting to the properties of the logical algebras, the relations among special types of fuzzy filters were given. The proofs were tedious in many literatures. The motivation of this paper is to give the simple general principle of studying the relations among fuzzy *t*-filters on residuated lattices. In contrast to proofs of particular results for concrete special types of fuzzy filters, proofs of those general theorems in this paper are simple. And the general principle can be applied to all the subvarieties of residuated lattices.

## 2. Preliminary

**Definition 1 (see [1, 25]).**

A residuated lattice is an algebra *L* = (*L*, ∧, ∨, ⊗, →, 0,1) such that for all *x*, *y*, *z* ∈ *L*,

1. (*L*, ∧, ∨, 0,1) is a bounded lattice;
2. (*L*, ⊗, 1) is a commutative monoid;
3. (⊗, →) forms an adjoint pair; that is, *x* ⊗ *y* ≤ *z* if and only if *x* ≤ *y* → *z*.

We denote *x* → 0 = *x* ^*^.

**Definition 2 (see [11, 25–30]).**

Let *L* be a residuated lattice. Then *L* is called

- an MTL-algebra if (*x* → *y*)∨(*y* → *x*) = 1 for all *x*, *y* ∈ *L* (prelinear axiom);
- an Rl-monoid if *x*∧*y* = *x* ⊗ (*x* → *y*) for all *x*, *y* ∈ *L* (divisible axiom);
- a Heyting algebra if *x* ⊗ *y* = *x*∧*y* for all *x*, *y* ∈ *L*, which is equivalent to an idempotent residuated lattice; that is, *x* = *x* ⊗ *x* = *x* ^2^ for *x* ∈ *L*;
- a regular residuated lattice if it satisfies double negation; that is, *x* ^**^ = *x* for *x* ∈ *L*;
- a BL-algebra if it satisfies both prelinear and divisible axioms;
- an MV-algebra if it is a regular Rl-monoid;
- a Gödel algebra if it is an idempotent BL-algebra;
- a Boolean algebra if it is an idempotent MV-algebra;
- a R0-algebra (NM algebra) if it satisfies prelinear axiom, double negation, and (*x* ⊗ *y* → 0)∨(*x*∧*y* → *x* ⊗ *y*) = 1.

**Definition 3 (see [25, 31, 32]).**

Let *L* be a residuated lattice. Then, a nonempty subset *F* of *L* is called a filter if

1. for all *x* ∈ *F* and *y* ∈ *L*, *x* ≤ *y* implies *y* ∈ *F*,
2. for all *x*, *y* ∈ *F*, *x* ⊗ *y* ∈ *F*.

**Definition 4 (see [5–11]).**

Let *F* be a filter of *L*. Then, *F* is called

- an implicative filter if *x* → *x* ^2^ ∈ *F* for all *x* ∈ *L*,
- a regular filter if *x* ^**^ → *x* ∈ *F* for all *x* ∈ *L*,
- a divisible filter if (*x*∧*y*)→(*x* ⊗ (*x* → *y*)) ∈ *F* for all *x*, *y* ∈ *L*,
- a prelinear filter if (*x* → *y*)∨(*y* → *x*) ∈ *F* for all *x*, *y* ∈ *L*,
- a Boolean filter if *x*∨*x* ^*^ ∈ *F* for all *x* ∈ *L*,
- a fantastic filter if (*y* → *x*)→(((*x* → *y*) → *y*) → *x*) ∈ *F* for all *x*, *y* ∈ *L*,
- an *n*-contractive filter if *x* ^*n*^ → *x* ^*n*+1^ ∈ *F* for all *x* ∈ *L*, where *x* ^*n*+1^ = *x* ^*n*^ ⊗ *x*, *n* ≥ 1.

**Remark 5 .**

On residuated lattices, *x* → (*y* → *z*) = *y* → (*x* → *z*) holds (see [31]). Using these properties, we have that *F* is a fantastic filter if ((*x* → *y*) → *y*)→((*y* → *x*) → *x*) ∈ *F*.

We now review some fuzzy concepts. A fuzzy set on residuated lattice is a function *μ* : *L* → [0,1]. For any *α* ∈ [0,1] and an arbitrary fuzzy set *μ*, we denote the set {*x* ∈ *L*∣*μ*(*x*) ≥ *α*} (i.e., the *α*-cut) by the symbol *μ*_*α*_.

**Definition 6 (see [10, 11]).**

A fuzzy set *μ* is a fuzzy filter on *L* if and only if it satisfies the following two conditions for all *x*, *y* ∈ *L*:

1. *μ*(*x* ⊗ *y*) ≥ min⁡{*μ*(*x*), *μ*(*y*)},
2. if *x* ≤ *y*, then *μ*(*x*) ≤ *μ*(*y*).

In the following, by the symbol $\overset{-}{x}$ we denote the abbreviation of *x*, *y*,…; that is, $\overset{-}{x}$ is a formal listing of variables used in a given content. By the term *t*, it is always meant as a term in the language of residuated lattices.

**Definition 7 (see [9]).**

Let *t* be an arbitrary term on the language of residuated lattices. A filter *F* on *L* is a *t*-filter if $t(\overset{-}{x})\in F$ for all $\overset{-}{x}\in L$.

**Definition 8 (see [23]).**

A fuzzy filter *μ* on *L* is called a fuzzy *t*-filter on *L*, if for all $\overset{-}{x}\in L$ it satisfies $\mu (t(\overset{-}{x}))=\mu (1)$.

**Example 9 (see [11]).**

Fuzzy Boolean filters are fuzzy *t*-filters for *t* equal to *x*∨*x* ^*^.

**Example 10 (see [11]).**

Fuzzy regular filters are fuzzy *t*-filters for *t* equal to *x* ^**^ → *x*.

**Example 11 (see [11]).**

Fuzzy fantastic filters are fuzzy *t*-filters for *t* equal to ((*x* → *y*) → *y*)→((*y* → *x*) → *x*).

**Remark 12 .**

Using the notion of fuzzy *t*-filter, fuzzy implicative filters are fuzzy *t*-filters for *t* equal to *x* → *x* ^2^. Fuzzy divisible filters are fuzzy *t*-filters for *t* equal to (*x*∧*y*)→(*x* ⊗ (*x* → *y*)) and so forth.

Let us assume that since now, *t* is an arbitrary term in the language of residuated lattices. We also use another useful convention: given a variety *B* of residuated lattices, we denote its subvariety given by the equation *t* = 1 by the symbol *B*[*t*]; we call this algebra *t*-algebra.

The next part of this paper concerns fuzzy quotient constructions. We recall some known results and constructions concerning fuzzy quotients residuated lattices.

**Theorem 13 (see [11]).**

Let *μ* be a fuzzy filter on *L* and *x*, *y* ∈ *L*. For any *z* ∈ *L*, we define *μ* ^*x*^ : *L* → [0,1], *μ* ^*x*^(*z*) = min⁡{*μ*(*x* → *z*), *μ*(*z* → *x*)}. Then, *μ* ^*x*^ = *μ* ^*y*^ if and only if *μ*(*x* → *y*) = *μ*(*y* → *x*) = *μ*(1).

**Theorem 14 (see [11]).**

Let *μ* be a fuzzy filter on *L* and *L*/*μ* : = {*μ* ^*x*^∣*x* ∈ *L*}. For any *μ* ^*x*^, *μ* ^*y*^ ∈ *L*/*μ*, we define

 
*μ* ^*x*^∧*μ* ^*y*^ = *μ* ^*x*∧*y*^,
 
*μ* ^*x*^∨*μ* ^*y*^ = *μ* ^*x*∨*y*^,
 
*μ* ^*x*^ ⊗ *μ* ^*y*^ = *μ* ^*x*⊗*y*^,
 
*μ* ^*x*^ → *μ* ^*y*^ = *μ* ^*x*→*y*^.

Then, *L*/*μ* = (*L*/*μ*, ∧, ∨, ⊗, →, *μ* ^0^, *μ* ^1^) is a residuated lattice called the fuzzy quotient residuated lattice.

**Theorem 15 (quotient characteristics [23]).**

Let *B* be a variety of residuated lattices and *L* ∈ *B*. Let *μ* be a fuzzy filter on *L*. Then, the fuzzy quotient *L*/*μ* belongs to *B*[*t*] if and only if *μ* is a fuzzy *t*-filter on *L*.

## 3. The General Principle of the Relation among Fuzzy *t*-Filters and Its Application

In the following, let *B* be a variety of residuated lattices. *L* ∈ *B* and *μ* is a fuzzy filter on *L*.

**Theorem 16 .**

Suppose that there are fuzzy *t*_1_-filter and fuzzy *t*_2_-filter on *L* and *B*[*t*_1_]⊆*B*[*t*_2_]. If *μ* is a fuzzy *t*_1_-filter, then *μ* is a fuzzy *t*_2_-filter.

**Proof**

*μ* is a fuzzy *t*_1_-filter ⇒*L*/*μ* ∈ *B*[*t*_1_]⇒*L*/*μ* ∈ *B*[*t*_2_]⇒*μ* is a fuzzy *t*_2_-filter.

**Theorem 17 .**

Suppose there are fuzzy *t*_1_-filter and fuzzy *t*_2_-filter on *L*. If *B*[*t*_1_] = *B*[*t*_2_], then *μ* is a fuzzy *t*_1_-filter if and only if *μ* is a fuzzy *t*_2_-filter.

**Proof**

*μ* is a fuzzy *t*_1_-filter ⇔*L*/*μ* ∈ *B*[*t*_1_]⇔*L*/*μ* ∈ *B*[*t*_2_]⇔*μ* is a fuzzy *t*_2_-filter.

**Remark 18 .**

The above results give the general principle of the relations among fuzzy *t*-filters. If we want to judge the relations among fuzzy *t*-filter, we only resort to the relations among *t*-algebras. Since the relations among *t*-algebras are known to us, we can easily obtain the relations among fuzzy *t*-filters.

**Theorem 19 .**

Let *L* be a residuated lattice. If *μ* is a fuzzy implicative filter, then *μ* is a fuzzy *n*-contractive filter.

**Proof**

It is obvious that *B*[*x* → *x* ^2^]⊆*B*[*x* ^*n*^ → *x* ^*n*+1^]. By Theorem 16, the result is clear.

**Lemma 20 (see [5, 27]).**

Let *L* be a residuated lattice. If *L* is a Heyting algebra, then *L* is an Rl-monoid.

**Lemma 21 (see [11]).**

Let *L* be a residuated lattice. Then the following are equivalent:

1. *L* is an MV-algebra;
2. (*x* → *y*) → *y* = (*y* → *x*) → *x*, ∀*x*, *y* ∈ *L*.

**Lemma 22 (see [25]).**

Let *L* be a residuated lattice. Then *L* is an MV-algebra if and only if *L* is a regular BL-algebra.

**Lemma 23 .**

Let *L* be a residuated lattice. Then the following are equivalent:

1. *L* is a Boolean algebra;
2. *x*∨*x* ^*^ = 1, ∀*x* ∈ *L*;
3. *L* is regular and idempotent.

**Proof**

(1)⇔(2) Reference [11], Proposition 2.10.

(1)⇒(3) If *L* is a Boolean algebra, then *L* is an idempotent MV-algebra. Thus, *L* is regular and idempotent.

(3)⇒(1) If *L* is regular and idempotent, then *L* is a regular Rl-monoid. Thus, *L* is an MV-algebra. Also, *L* is idempotent; therefore, *L* is a Boolean algebra.

**Lemma 24 .**

Let *L* be a residuated lattice. Then the following are equivalent:

1. *L* is a Boolean algebra;
2. *L* is an idempotent R0-algebra.

**Proof**

(1)⇒(2) If *L* is a Boolean algebra, then *L* is a R0-algebra ([30], Example 8.5.1) and *L* is idempotent.

(2)⇒(1) Suppose *L* is an idempotent R0-algebra. Since *L* is a R0-algebra, then *L* is regular. By Lemma 23, we have that *L* is a Boolean algebra.

**Lemma 25 .**

Let *L* be a residuated lattice. *t*_1_ and *t*_2_ are arbitrary terms on *L*. Then, *B*[*t*_1_ ⊗ *t*_2_] = *B*[*t*_1_]∩*B*[*t*_2_].

**Proof**

*L* ∈ *B*[*t*_1_ ⊗ *t*_2_]⇔*L* ∈ *B* and *t*_1_ ⊗ *t*_2_ = 1⇔*L* ∈ *B*; *t*_1_ = 1 and *t*_2_ = 1⇔*L* ∈ *B*[*t*_1_]∩*B*[*t*_2_].

**Theorem 26 .**

Let *L* be a residuated lattice. Then, *μ* is a fuzzy Boolean filter if and only if *μ* is both a fuzzy regular and a fuzzy implicative filter.

**Proof**

*μ* is a fuzzy Boolean filter ⇔*L*/*μ* ∈ *B*[*x*∨*x* ^*^]⇔*L*/*μ* ∈ *B*[(*x* ^**^ → *x*)⊗(*x* → *x* ^2^)]⇔*L*/*μ* ∈ *B*[*x* ^**^ → *x*]∩*B*[*x* → *x* ^2^]⇔*L*/*μ* ∈ *B*[*x* ^**^ → *x*] and *L*/*μ* ∈ *B*[*x* → *x* ^2^]⇔*μ* is both a fuzzy regular and a fuzzy implicative filter.

**Remark 27 .**

Using the same method, we can easily obtain the following results.

**Theorem 28 .**

Let *L* be a residuated lattice. Then

1. *μ* is a fuzzy Boolean filter if and only if *μ* is a fuzzy fantastic and fuzzy implicative filter;
2. *μ* is a fuzzy fantastic filter if and only if *μ* is a fuzzy regular and fuzzy divisible filter;
3. every fuzzy implicative filter is a fuzzy divisible one;
4. if *μ* is a fuzzy prelinear filter, then *μ* is a fuzzy fantastic filter if and only if *μ* is both a fuzzy regular and a fuzzy divisible filter;
5. if *μ* is a fuzzy Boolean filter, then *μ* is a fuzzy *n*-contractive filter.

**Remark 29 .**

The notion of fuzzy *t*-filter and the general principle are not only applicable on residuated lattices, but also even transferable to all their subvarieties. Taking advantage of the relations among *t*-algebras, we can easily obtain the following results.

**Theorem 30 .**

Let *L* be a Boolean-algebra. Then the fuzzy prelinear filter, fuzzy fantastic filter, fuzzy divisible filter, fuzzy regular filter, and fuzzy *n*-contractive and fuzzy Boolean filter coincide.

**Theorem 31 .**

Let *L* be an MV-algebra. Then

1. *μ* is a fuzzy Boolean filter if and only if *μ* is a fuzzy implicative filter;
2. the fuzzy prelinear filter, fuzzy fantastic filter, fuzzy divisible filter, and fuzzy regular filter coincide.

**Theorem 32 .**

Let *L* be a Gödel-algebra. Then

1. *μ* is a fuzzy Boolean filter if and only if *μ* is a fuzzy regular filter if and only if *μ* is a fuzzy fantastic filter;
2. the fuzzy prelinear filter, fuzzy divisible filter, fuzzy *n*-contractive filter, and fuzzy implicative filter coincide.

**Theorem 33 .**

Let *L* be a BL-algebra; then

1. *μ* is a fuzzy Boolean filter if and only if *μ* is both a fuzzy implicative and a fuzzy regular filter;
2. *μ* is a fuzzy Boolean filter if and only if *μ* is both a fuzzy implicative and a fuzzy fantastic filter;
3. *μ* is a fuzzy fantastic filter if and only if *μ* is a fuzzy regular filter;
4. the fuzzy prelinear filter and fuzzy divisible filter coincide.

**Theorem 34 .**

Let *L* be an MTL-algebra. Then,

1. *μ* is a fuzzy Boolean filter if and only if *μ* is both a fuzzy implicative and a fuzzy regular filter;
2. *μ* is a fuzzy Boolean filter if and only if *μ* is both a fuzzy implicative and a fuzzy fantastic filter;
3. *μ* is a fantastic filter if and only if *μ* is a regular and divisible filter;
4. if *μ* is a fuzzy implicative filter, then *μ* is a fuzzy divisible filter.

**Theorem 35 .**

Let *L* be a Heyting-algebra. Then

1. *μ* is a fuzzy Boolean filter if and only if *μ* is a fuzzy regular filter if and only if *μ* is a fuzzy fantastic filter;
2. the fuzzy implicative filter, fuzzy divisible filter, and fuzzy *n*-contractive filter coincide.

**Theorem 36 .**

Let *L* be a R0-algebra; then

1. *μ* is a fuzzy Boolean filter if and only if *μ* is a fuzzy implicative filter;
2. every fuzzy Boolean filter is a fuzzy fantastic filter;
3. *μ* is a fuzzy fantastic filter if and only if *μ* is a fuzzy divisible filter;
4. the fuzzy prelinear filter and fuzzy regular filter coincide;
5. every fuzzy implicative filter is a fuzzy divisible one.

**Theorem 37 .**

Let *L* be a regular residuated lattice. Then

1. *μ* is a fuzzy Boolean filter if and only if *μ* is a fuzzy implicative filter;
2. *μ* is a fuzzy fantastic filter if and only if *μ* is a fuzzy divisible filter;
3. every fuzzy Boolean filter is a fuzzy fantastic filter;
4. every fuzzy implicative filter is a fuzzy divisible one.

**Theorem 38 .**

Let *L* be a Rl-monoid. Then

1. *μ* is a fuzzy Boolean filter if and only if *μ* is a fuzzy implicative and fuzzy fantastic filter;
2. *μ* is a fuzzy Boolean filter if and only if *μ* is a fuzzy implicative and fuzzy regular filter;
3. *μ* is a fuzzy fantastic filter if and only if *μ* is a fuzzy regular filter;
4. every fuzzy implicative filter is a fuzzy divisible one.

## References

[1] Ward M., Dilworth R. P. (1939). Residuated lattices. *Transactions of the American Mathematical Society*.

[2] Höhle U. (1995). *Commutative Residuated 1-Monoids, Non-Classical Logics and Their Applications to Fuzzy Subsets*.

[3] Idziak P. (1984). Lattice operation in BCK-algebras. *Mathematica Japonica*.

[4] Buşneag D., Piciu D. (2014). Some types of filters in residuated lattices. *Soft Computing*.

[5] Cretan R., Jeflea A. (2006). On the lattice of congruence filters of a residuated lattice. *Annals of the University of Craiova Mathematics and Computer Science*.

[6] van Gasse B., Deschrijver G., Cornelis C., Kerre E. E. (2010). Filters of residuated lattices and triangle algebras. *Information Sciences*.

[7] Kondo M. (2011). Characterization of some types of filters in commutative residuated lattices. *Far East Journal of Mathematical Sciences (FJMS)*.

[8] Ma Z. M., Hu B. Q. (2014). Characterizations and new subclasses of τ-filters in residuated lattices. *Fuzzy Sets and Systems*.

[9] Víta M. (2014). Why are papers about filters on residuated structures (usually) trivial?. *Information Sciences*.

[10] Zhang X. H., Zhou H. J., Mao X. Y. (2014). IMTL(MV)-filters and fuzzy IMTL(MV)-filters of residuated lattices. *Journal of Intelligent and Fuzzy Systems*.

[11] Zhu Y. Q., Xu Y. (2010). On filter theory of residuated lattices. *Information Sciences*.

[12] Zadeh L. A. (1965). Fuzzy sets. *Information and Computation*.

[13] Xu Y., Qin K. Y. (1995). Fuzzy lattice implication algebras. *Journal of Southwest Jiaotong University*.

[14] Jun Y. B. (2001). Fuzzy positive implicative and fuzzy associative filters of lattice implication algebras. *Fuzzy Sets and Systems*.

[15] Jun Y. B., Song S. Z. (2002). On fuzzy implicative filters of lattice implication algebras. *Journal of Fuzzy Mathematics*.

[16] Jun Y. B., Song S. Z. (2003). Fuzzy *n*-fold positive implicative filters in lattice implication algebras. *Journal of Applied Mathematics and Computing*.

[17] Jun Y. B., Song S. Z. (2004). On fuzzy fantastic filters of lattice implication algebras. *Journal of Applied Mathematics & Computing*.

[18] Jun Y. B., Xu Y., Zhang X. H. (2005). Fuzzy filters of MTL-algebras. *Information Sciences*.

[19] Kim K. H., Zhang Q., Jun Y. B. (2002). On fuzzy filters of MTL-algebras. *Journal of Fuzzy Mathematics*.

[20] Lianzhen L., Kaitai L. (2005). Fuzzy implicative and Boolean filters of *R*
_0_ algebras. *Information Sciences*.

[21] Liu L., Li K. (2005). Fuzzy filters of BL-algebras. *Information Sciences*.

[22] Lianzhen L., Kaitai L. (2005). Fuzzy Boolean and positive implicative filters of BL-algebras. *Fuzzy Sets and Systems*.

[23] Víta M. (2014). Fuzzy t-filters and their properties. *Fuzzy Sets and Systems*.

[24] Zhan J., Jun Y. B., Kim H. S. (2014). Some types of falling fuzzy filters of BL-algebras and its applications. *Journal of Intelligent and Fuzzy Systems*.

[25] Hájek P. (1998). *Metamathematics of Fuzzy Logic*.

[26] Esteva F., Godo L. (2001). Monoidal t-norm based logic: towards a logic for left-continuous t-norms. *Fuzzy Sets and Systems*.

[27] Haveshki M., Mohamadhasani M. (2012). Extended filters in bounded commutative Rl-monoids. *Soft Computing*.

[28] Muresan C. (2010). Dense elements and classes of residuated lattices. *Bulletin Mathematique de la Societe des Sciences Mathematiques de Roumanie*.

[29] Pei D. (2003). On equivalent forms of fuzzy logic systems NM and IMTL. *Fuzzy Sets and Systems*.

[30] Wang G. J. (2000). *Non-Classical Mathematical Logic and Approximate Reasoning*.

[31] Kowalski T., Ono H. (2001). *Residuated Lattices: An Algebraic Glimpse at Logic without Contraction*.

[32] Turunen E. (1999). *Mathematics behind Fuzzy Logic*.

